# Segmental Trisomy of Mouse Chromosome 17: Introducing an Alternative Model of Down’s Syndrome

**DOI:** 10.1002/cfg.334

**Published:** 2003-12

**Authors:** Jiri Forejt, Tomáš Vacík, Soňa Gregorová

**Affiliations:** Institute of Molecular Genetic Academy of Sciences of the Czech Republic and Center of Integrated Genomics Videnska 1083 Prague 124 20 Prague 4 Czech Republic

## Abstract

All of the mouse models of human trisomy 21 syndrome that have been studied so far are based on segmental trisomies, encompassing, to a varying extent, distal chromosome 16. Their comparison with one or more unrelated and non-overlapping segmental trisomies may help to distinguish the effects of specific triplicated genes from the phenotypes caused by less specific developmental instability mechanisms. In this paper, the Ts43H segmental trisomy of mouse chromosome 17 is presented as such an alternative model. The trisomy stretches over 32.5 Mb of proximal chromosome 17 and includes 486 genes. The triplicated interval carries seven blocks of synteny with five human chromosomes. The block syntenic to human chromosome 21 contains 20 genes.


**Comparative and Functional Genomics**
*Comp Funct Genom* 2003; **4**: 647–652.
Published online in Wiley InterScience (www.interscience.wiley.com). **DOI:** 10.1002/cfg.334

**Conference Review**

### Segmental trisomy of mouse chromosome 17: introducing an alternative model of Down's syndrome

Jiri Forejt*, Tomáš Vacík and Soňa Gregorová
*Institute of Molecular Genetics, Academy of Sciences of the Czech Republic and Center of Integrated Genomics, Prague, Czech Republic*

**Correspondence to:
Jiri Forejt, Institute of Molecular Genetics, Academy of Sciences of the Czech Republic and Center of Integrated Genomics, Prague, Czech Republic, Videnska 1083, 124 20 Prague 4, Czech Republic.
E-mail: jforejt@biomed.cas.cz*

Received: 19 August 2003
Revised: 9 September 2003
Accepted: 9 September 2003

### Abstract

**All of the mouse models of human trisomy 21 syndrome that have been studied so far are based on segmental trisomies, encompassing, to a varying extent, distal chromosome 16. Their comparison with one or more unrelated and non-overlapping segmental trisomies may help to distinguish the effects of specific triplicated genes from the phenotypes caused by less specific developmental instability mechanisms. In this paper, the Ts43H segmental trisomy of mouse chromosome 17 is presented as such an alternative model. The trisomy stretches over 32.5 Mb of proximal chromosome 17 and includes 486 genes. The triplicated interval carries seven blocks of synteny with five human chromosomes. The block syntenic to human chromosome 21 contains 20 genes. Copyright © 2003 John Wiley & Sons, Ltd.**

**Keywords:** segmental aneuploidy; Down's syndrome; Ts43H; trisomy; gene dosage; imprinting; mouse

### Introduction

The presence of three copies of an autosome in the mouse genome seriously impairs embryonic development and almost invariably results in embryonic lethality (Gropp *et al*., 1983; Hernandez and Fisher, 1999). In humans, one-half of all recognized spontaneous abortions are associated with aneuploidy, half of these being due to a trisomy. Only three human trisomies are compatible with partial postnatal survival (but with extremely affected development). The trisomy 21, known as Down's syndrome (DS), is the most frequent cause of mental retardation with an incidence of 1 per 700 newborns. Only 10% of foetuses with trisomy 21 survive to term (Epstein, 1988). Trisomies 18 and 13, causing Edwards and Patau syndromes, are also semi-viable but display even more severe clinical features (Nicolaidis and Petersen, 1998).

Two hypotheses, which are not mutually exclusive, were proposed to explain why an extra copy of an otherwise normal autosome has such devastating effects on development. According to the 'dosage-sensitive genes' hypothesis, the trisomic region carries a subset of genes, which when triplicated are responsible for all pathological features (Korenberg *et al*., 1994). An extra copy of a particular dosage-sensitive gene is assumed to account for a particular phenotypic trait in a trisomic organism. According to the 'developmental instability' hypothesis, the massive overexpression of triplicated genes results in a non-specific breakdown of the gene regulatory networks during development and consequently in chaos of fine tuning of developmental pathways (Pritchard and Kola 1999; Shapiro 1997). This hypothesis questions attempts to link particular triplicated genes to particular traits. Instead, it predicts that an extra copy of any autosomal segment of sufficient length, with a sufficient number of expressed genes, is likely to cause a developmental breakdown.

Because of its high incidence and clinical importance, almost all papers on the molecular nature of human trisomies have been focused on DS, at the identification of critical chromosomal regions or on attempts to positionally clone candidate genes

Copyright © 2003 John Wiley & Sons, Ltd.

for particular pathological traits. Admittedly, in spite of extensive experimental efforts, not a single candidate gene, when used as a transgene in the mouse model, has fully recapitulated the relevant trait. However, when using the extra copy of a chromosomal segment in the mouse segmental trisomy models, it was possible to recapitulate several major traits including cognitive defects, characteristic skull dysgenesis or cerebellar maldevelopment (Reeves *et al.*, 2001).

To evaluate which phenotypic feature or gene expression anomaly can be attributed to the developmental instability hypothesis and which is compatible with the idea of dosage-sensitive genes, it may be helpful to use different mouse trisomic models that replicate human trisomy 21. By searching for features that are common to two unrelated and non-overlapping mouse trisomies, the specificity of the observed phenotypes can be evaluated. Here we introduce the Ts43H segmental trisomy that triplicates the proximal part of the mouse chromosome 17, from the centromere to the *H2-K* gene, which could be used for such a purpose. Because 20 out of 486 triplicated genes within the Ts43H trisomic interval are orthologous to genes on human chromosome 21, we start with a brief description of syntenies between human chromosome 21 and the mouse genome and with a comparison of Ts43H to other viable mouse segmental trisomies.

### Regions of synteny between human chromosome 21 and the mouse genome

The total length of human chromosome 21 is 46.98 Mb, representing 1.5% of the human genome and carrying 1.1% (273/24261) of all human genes (according to the Human Genome Browser, Release 16.33.1: **http://www.ensembl.org/Homo_sapiens/**). Following the concept of the dosage-sensitive gene hypothesis, the ideal mouse model of human DS would carry three copies of all of the mouse genes with orthologues on human chromosome 21. However, during 75 million years of separate evolution, the human and mouse genomes have been reshuffled by chromosomal rearrangements so that, at present, the regions of synteny with human chromosome 21 can be found on three mouse chromosomes (27.9 Mb on chromosome 16, 2.9 Mb on chromosome 10 and 1.6 Mb on chromosome 17: Figure 1; Table 1).

The syntenic region on mouse chromosome 16 has been studied thoroughly using the segmental trisomies Ts65Dn, Ts1Cje and their derivatives (Dierssen *et al.*, 2001; Kola and Hertzog, 1998; Reeves *et al.*, 2001). A viable mouse trisomy of the A1–C1 bands of chromosome 10 originated from the T(10;13)199H translocation (Eicher and Washburn, 1977; Lyon and Meredith, 1966). The available mapping data of the T199H breakpoint on chromosome 10 (Beechey and Evans, 1996) suggest that the Ts199H trisomic region most likely encompasses the block of genes syntenic with human chromosome 21. The interval on mouse chromosome 17 syntenic to human chromosome 21 is triplicated in the Ts43H segmental trisomy, which is a derivative the of T(16;17)43H reciprocal translocation.

### Origin and characteristics of the T(16;17)43H translocation and the Ts43H trisomy

The reciprocal translocation between chromosomes 16 and 17, T(16;17)43H, was generated by the irradiation of sperm cells of a (C3H × 101)$F_1$ hybrid

**Table 1.** Length and gene content of mouse chromosomal regions (chromosomes 10, 16 and 17) syntenic to human chromosome 21*

| Mouse synteny to human chromosome 21 | Human Ensembl map (Mbp) | Length of human synteny (Mbp) | Number of human genes | Mouse Ensembl map (Mbp) | Length of mouse synteny (Mbp) | Number of mouse genes |
|---|---|---|---|---|---|---|
| Chromosome 10 | 44.0–46.9 | 2.9 | 59 | 76.0–78.3 | 2.3 | 47 |
| Chromosome 16 | 14.4–42.3 | 27.9 | 151 | 76.0–98.6 | 22.6 | 172 |
| Chromosome 17 | 42.4–44.0 | 1.6 | 25 | 29.7–30.8 | 1.1 | 20 |
| Total | | 32.4 | 235 | | 26 | 239 |

* Based on Mouse Genome Assembly NCBI 30 (**http://www.ensembl.org/Mus_musculus/**).

Copyright © 2003 John Wiley & Sons, Ltd. 

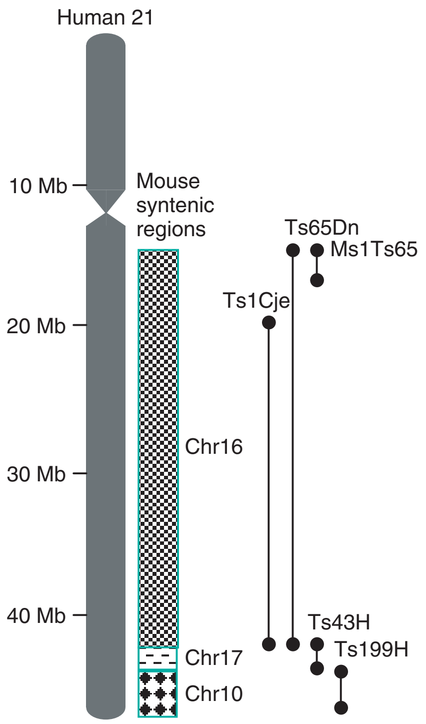

**Figure 1.** Regions of synteny between human chromosome 21 and the mouse genome. The intervals of mouse chromosome 16 triplicated in segmental trisomies Ts65Dn, Ts1Cje and MsTs65Dn are indicated. The trisomies T43H and Ts199H have not been used so far for analysis of genes corresponding to human chromosome 21

male (Searle *et al.*, 1974). The translocation causes the complete arrest of spermatogenesis in males, but T43H/+ female heterozygotes are fertile. Male fertility can be restored by combining the T43H and Rb(16;17)Bnr translocations. Using this ploy it was possible to generate T43H/T43H homozygous stock, which proved to be fertile in both sexes (Gregorova *et al.*, 1981).

Viable Ts43H mice with segmental trisomy of the proximal part of chromosome 17 arise as a product of adjacent II disjunction in female meiosis of T43H translocation heterozygotes (Forejt, 2002; Forejt *et al.*, 1980). The frequency of viable trisomics in the progeny varies, depending on the genetic background, from 0% to 20%, and can be enhanced by the presence of a *t*-haplotype (Capkova *et al.* 1986; Gregorova *et al.*, 1981). The Ts43H trisomic mice have 40 chromosomes but, in contrast to euploid wild-type mice, one chromosome 16 carries an extra copy of the proximal region of chromosome 17 on the top of its centromeric heterochromatin (Figure 2). The Ts43H trisomics can be identified by cytogenetic methods, detecting a unique interstitial C-band on the $17^{16}$ marker chromosome and the simultaneous absence of the small $16^{17}$ translocation product. A single nucleotide primer extension method (SnuPE) was developed for DNA genotyping the Ts43H trisomics (Vacik and Forejt, 2003) and more recently a diagnostic microsatellite marker was found with three different alleles in the three chromosomes engaged in the segmental trisomy (Vacik, unpublished results). The translocation breakpoint on chromosome 17 was shown to be tightly linked, but proximal, to the *H2K* gene (Forejt *et al.*, 1980).

### Regions of synteny between the Ts43H segmental trisomy and the human genome

The Ts43H triplicated region stretches from the centromere of chromosome 17 to the T43H translocation break. It is 32.5 Mb long and carries 486

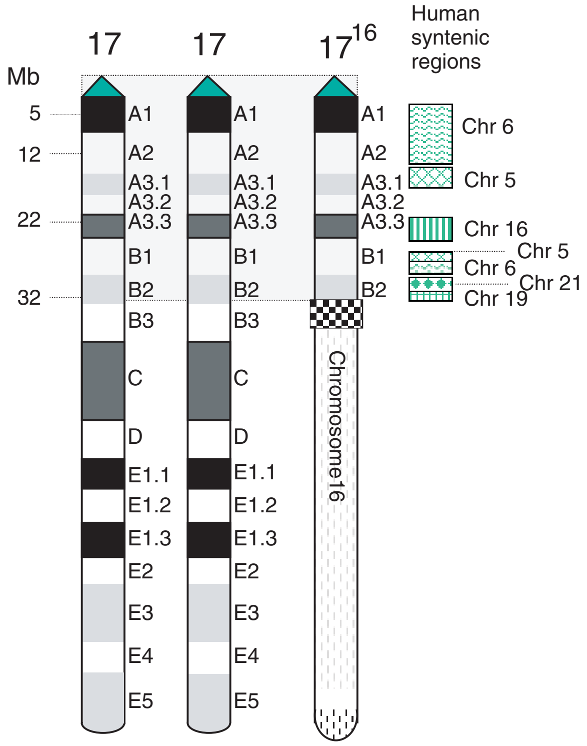

**Figure 2.** The chromosomes involved in the Ts43H trisomy and the corresponding human syntenic regions. The triplicated part of chromosome 17 is framed

Copyright © 2003 John Wiley & Sons, Ltd. 

genes, representing 1.3% of mouse genome and 1.9% of mouse genes. This region of chromosome 17 overlaps with the *T–t*-haplotype complex, the naturally occurring variant form of chromosome 17 composed of four adjacent inversions and a series of lethal mutations (Hammer *et al.*, 1989; Lyon, 2000). The unstable character of the region is also obvious when it is compared with the human genome. The Ts43H interval shows seven blocks of synteny with five human chromosomes (Figure 2; Table 2). The block syntenic to human chromosome 21 carries 20 genes (Table 3) and their expression analysis can be used to complete the set of existing mouse DS models. The triplicated region includes 16 genes with orthologues in the list of human disease genes (Table 4) in the Online Mendelian Inheritance in Man (OMIM) database (**http://www.ncbi.nlm.nih.gov/Omim/**).

### The imprinted genes within the triplicated region

Mouse chromosome 17 carries four imprinted genes, insulin-like growth factor 2 receptor (*Igf2r*),

**Table 2.** Length and gene content of regions of synteny between the Ts43H trisomic interval of mouse chromosome17 and human chromosomes 5, 6, 16, 19 and 21*

| Human synteny to Ts43H | Mouse Ensembl map (Mbp) | Length of mouse synteny (Mbp) | Number of mouse genes | Human Ensembl map (Mbp) | Length of human synteny (Mbp) | Number of human genes |
|---|---|---|---|---|---|---|
| Chromosome 6 | 3.0–13.9 | 10.9 | 108 | 150.3–170.3 | 20.0 | 113 |
| Chromosome 5 | 14.2–16.2 | 2.0 | 14 | 96.2–98.4 | 2.2 | 12 |
| Chromosome 16 | 22.2–25.0 | 2.8 | 145 | 0.2–3.2 | 3.0 | 136 |
| Chromosome 5 | 25.0–25.6 | 0.6 | 10 | 171.9–172.6 | 0.7 | 10 |
| Chromosome 6 | 25.6–29.7 | 4.1 | 79 | 33.4–39.1 | 5.7 | 75 |
| Chromosome 21 | 29.7–30.8 | 1.1 | 20 | 42.4–44.0 | 1.6 | 25 |
| Chromosome 19 | 30.8–31.3 | 0.5 | 8 | 15.1–15.6 | 0.5 | 12 |
| Total | | 22.0 | 384 | | 33.7 | 383 |

* Based on Mouse Genome Assembly NCBI 30 (**http://www.ensembl.org/Mus_musculus/**).

**Table 3.** Map coordinates of mouse genes from the Ts43H trisomic region with orthologues on human chromosome21

| Ensembl gene ID | Gene symbol | Mouse Ensembl map (Mb) | Mouse MGI map (cM) | Human Ensembl map (Mb) |
|---|---|---|---|---|
| ENSMUSG00000024030 | Abcg1 | 29.77 | — | 42.53 |
| ENSMUSG00000024029 | Tff3 | 29.84 | 17.0 | 42.63 |
| ENSMUSG00000024028 | Tff2 | 29.86 | 17.0 | 42.66 |
| ENSMUSG00000024032 | Tff1 | 29.88 | 17.0 | 42.68 |
| ENSMUSG00000024034 | Tmprss3 | 29.90 | — | 42.69 |
| ENSMUSG00000042345 | (UBASH3A) | 29.92 | — | 42.72 |
| ENSMUSG00000024033 | Tsga2 | 29.97 | 13.25 | 42.79 |
| ENSMUSG00000024036 | (SLC37A1) | 30.00 | — | 42.83 |
| ENSMUSG00000041119 | Pde9a | 30.10 | — | 42.97 |
| ENSMUSG00000024037 | Wdr4 | 30.21 | — | 43.16 |
| ENSMUSG00000024038 | 1500032D16Rik | 30.23 | — | 43.20 |
| ENSMUSG00000013562 | 4833413E03Rik | 30.27 | — | — |
| ENSMUSG00000006705 | Pknox1 | 30.29 | — | 43.29 |
| ENSMUSG00000024039 | Cbs | 30.32 | 17.4 | 43.37 |
| ENSMUSG00000046684 | — | 30.35 | — | — |
| ENSMUSG00000024040 | U2af1 | 30.36 | — | 43.41 |
| ENSMUSG00000024041 | Cryaa | 30.39 | 17.4 | 43.48 |
| ENSMUSG00000024042 | Snf1lk | 30.55 | 18.18 | 43.69 |
| ENSMUSG00000002076 | Hsf2bp | 30.65 | — | 43.81 |
| ENSMUSG00000002070 | 2600005C20Rik | 30.75 | — | 43.94 |

Copyright © 2003 John Wiley & Sons, Ltd.

**Table 4.** List of human disease genes (OMIM) with mouse orthologues in the Ts43H trisomic region

| Chromosome No. | Gene symbol | Disease OMIM ID | Disease description |
|---|---|---|---|
| 5 | NKX2-5 | 600584 | Atrial septal defect with atrioventricular conduction defects |
| 6 | ACAT2 | 100678 | ACAT2 deficiency |
| 6 | IGF2R | 147280 | Hepatocellular carcinoma |
| 6 | LPA | 152200 | Coronary artery disease, susceptibility to |
| 6 | PLG | 173350 | Plasminogen Tochigi's disease |
| 6 | PARK2 | 602544 | Parkinson's disease, juvenile, type 2 |
| 6 | HMGA1 | 600701 | Lipoma |
| 6 | TULP1 | 602280 | Retinitis pigmentosa |
| 6 | MTCH1 | 176801 | Gaucher's disease, variant form |
| 16 | HAGH | 138760 | Glyoxalase II deficiency |
| 16 | TSC2 | 191092 | Tuberous sclerosis-2 |
| 16 | PKD1 | 601313 | Polycystic kidney disease, adult type 1 |
| 19 | NOTCH3 | 600276 | Cerebral arteriopathy with subcortical infarcts and leukoencephalopathy |
| 21 | TMPRSS3 | 601072 | Deafness, autosomal recessive |
| 21 | CBS | 236200 | Homocystinuria, B6-responsive and non-responsive types |
| 21 | CRYAA | 123580 | Cataract, congenital, autosomal dominant |

insulin-like growth factor 2 receptor antisense RNA (*Air*) and solute carrier family 22 members 2 and 3 (*Slc22a2* and *Slc22a3*). All of them are in one imprinted domain within the Ts43H region and all but *Air* are paternally repressed. Recently, a large-scale expression profiling of 9.5 dpc parthenogenote and androgenote mouse embryos revealed 34 transcripts within the Ts43H region that are either imprinted or regulated by imprinted genes (Nikaido *et al.*, 2003). The monoallelic expression of imprinted genes can be viewed as a special case of gene-dosage regulation (Vacik and Forejt, 2003), which makes them potential candidates for dosage-sensitive genes within the trisomic regions. The analysis of expression of the *Igf2r* gene in Ts43H trisomics showed that the paternal repression and maternal transcription of *Igf2r* was not affected in trisomics, regardless of whether the extra copy was of paternal or maternal origin. Moreover, mRNA levels of the *Igf2r* gene did not differ in mouse embryos with one or two active copies of the gene, indicating some kind of dosage compensation mechanism controlling this triplicated imprinted gene (Vacik and Forejt, 2003).

### Future prospects of the Ts43H trisomy as a model of aneuploidy syndromes

Recent expression profiling experiments have revealed global upregulation of chromosome 21 gene expression in foetal DS brains (Mao *et al.*, 2003) and general misregulation of genes outside the trisomy in cultured human foetal cells (FitzPatrick *et al.*, 2002) as well as in the cerebellar transcriptome of adult Ts65Dn trisomic mice (Saran *et al.*, 2003). These data strongly suggest, but do not prove, that the global destabilization of gene regulation plays an important role in the phenotypic manifestation of such aneuploidies.

The Ts43H trisomy can be used for experimental testing of both hypotheses, by searching for dosage-sensitive genes and by comparing it with unrelated trisomies, such as Ts65Dn or Ts199H. Analysis of Ts43H mice has just begun and the first results on learning deficit and expression profiling in brains are encouraging (Vacik, Orth, Gregorova, Strnad, Bures and Forejt, manuscript in preparation). Comparisons between two or more viable segmental trisomies may bring new answers to some of the old questions provoked by the two previously mentioned hypotheses, including:

- Are there any phenotypic features or gene expression patterns common to unrelated segmental trisomies that are not observed in wild-type siblings? To assess the phenotypes properly, the complex examination of trisomics and their wild-type littermates should be done following established programs, such as those of Mouse Phenome project (Paigen and Eppig, 2000) (**http://aretha.jax.org/pub-cgi/phenome/mpdcgi?rtn=docs/home**) or the 'Mouse Clinic' (**http://www.gsf.de/ieg/gmc/index.html**).
- Why do a fraction of segmental trisomics die before term? Is there any genetic or epigenetic difference between the prospectively viable and prospectively lethal trisomic foetuses? If there is such a difference, is it shared by unrelated trisomies? What is the nature of the abnormal phenotypic variability observed in segmental

Copyright © 2003 John Wiley & Sons, Ltd. 

aneuploidies? Can we find modifier genes that would suppress some of the pathological features associated with segmental aneuploidies?

- Is it the size of the triplicated segment, or its specific gene content, that determines particular pathological features? A combination of the Ts43H trisomy with various deletions created in the same chromosomal interval in Dr Schimenti's laboratory (Chao *et al.*, 2003) could be instrumental in this respect.

These are some of the problems that can be studied by comparing unrelated mouse models of segmental trisomies, utilizing all of the advantages provided by current mouse genetic and genomic methodologies.

### Acknowledgements

We thank Zdenek Trachtulec John Novotney for useful comments on the manuscript. The work in the laboratory was supported by the Grant Agency of the Czech Republic, Grant No. 204/98/K015. J. F. is supported as an International Scholar of the Howard Hughes Medical Institute.

### References

Beechey C, Evans E. 1996. Chromosomal variants. In *Genetic Variants and Strains of the Laboratory Mouse*, Lyon M, Rastan S, Brown S (eds). Oxford University Press: Oxford; 1469–1470.

Capkova J, Gregorova S, Forejt J. 1986. Recessive lethal t haplotypes increase the frequency of the partial trisomy of chromosome 17 (including the $T-t$ complex) among offspring of T(16;17)43H female mice. *Folia Biol (Praha)* **32**: 26–35.

Dierssen M, Fillat C, Crnic L, *et al.* 2001. Murine models for Down's syndrome. *Physiol Behav* **73**: 859–871.

Eicher E, Washburn L. 1977. Research News. *Mouse News Lett* **56**: 43.

Epstein CJ. 1988. Mechanisms of the effects of aneuploidy in mammals. *Annu Rev Genet* **22**: 51–75.

FitzPatrick DR, Ramsay J, McGill NI, *et al.* 2002. Transcriptome analysis of human autosomal trisomy. *Hum Mol Genet* **11**: 3249–3256.

Forejt J. 2002. Nondisjunction. In *Encyclopedia of Genetics*. Brenner S, Miller J (eds.). Academic Press: San Diego; 1345–1347.

Forejt J, Capkova J, Gregorova S. 1980. T(16 : 17)43H translocation as a tool in analysis of the proximal part of chromosome 17 (including $T-t$ gene complex) of the mouse. *Genet Res* **35**: 165–177.

Gregorova S, Baranov V, Forejt J. 1981. Partial trisomy (including $T-t$ gene complex) of the chromosome 17 of the mouse. The effect on male fertility and the transmission to progeny. *Folia Biol (Praha)* **27**: 171–177.

Gropp A, Winking H, Herbst EW, Claussen CP. 1983. Murine trisomy: developmental profiles of the embryo, and isolation of trisomic cellular systems. *J Exp Zool* **228**: 253–269.

Hammer M, Schimenti J, Silver L. 1989. Evolution of mouse chromosome 17 and the origin of inversions associated with *t* haplotypes. *Proc Natl Acad Sci USA* **86**: 3261–3265.

Hernandez D, Fisher EM. 1999. Mouse autosomal trisomy: two's company, three's a crowd. *Trends Genet* **15**: 241–247.

Chao HH, Mentzer SE, Schimenti JC, You Y. 2003. Overlapping deletions define novel embryonic lethal loci in the mouse *t* complex. *Genesis* **35**: 133–142.

Kola I, Hertzog PJ. 1998. Down's syndrome and mouse models. *Curr Opin Genet Dev* **8**: 316–321.

Korenberg JR, Chen XN, Schipper R, *et al.* 1994. Down's syndrome phenotypes: the consequences of chromosomal imbalance. *Proc Natl Acad Sci USA* **91**: 4997–5001.

Lyon M, Meredith R. 1966. Autosomal translocations causing male sterility and viable aneuploidy in the mouse. *Cytogenetics* **5**: 335–354.

Lyon MF. 2000. An answer to a complex problem: cloning the mouse *t*-complex responder. *Mamm Genome* **11**: 817–819.

Mao R, Zielke CL, Ronald Zielke H, Pevsner J. 2003. Global upregulation of chromosome 21 gene expression in the developing Down's syndrome brain. *Genomics* **81**: 457–467.

Nicolaidis P, Petersen M. 1998. Origin and mechanisms of non-disjunction in human autosomal trisomies. *Hum Reprod* **13**: 313–319.

Nikaido I, Saito C, Mizuno Y, *et al.* 2003. Discovery of imprinted transcripts in the mouse transcriptome using large-scale expression profiling. *Genome Res* **13**: 1402–1409.

Paigen K, Eppig JT. 2000. A mouse phenome project. *Mamm Genome* **11**: 715–717.

Pritchard MA, Kola I. 1999. The 'gene dosage effect' hypothesis vs. the 'amplified developmental instability' hypothesis in Down's syndrome. *J Neur Transm Suppl* **57**: 293–303.

Reeves RH, Baxter LL, Richtsmeier JT. 2001. Too much of a good thing: mechanisms of gene action in Down's syndrome. *Trends Genet* **17**: 83–88.

Saran NG, Pletcher MT, Natale JE, *et al.* 2003. Global disruption of the cerebellar transcriptome in a Down's syndrome mouse model. *Hum Mol Genet* **12**: 2013–2019.

Searle AG, Ford CE, Evans EP, *et al.* 1974. The induction of translocations in mouse spermatozoa. I. Kinetics of dose–response with acute X-irradiation. *Mutat Res* **22**: 157–174.

Shapiro BL. 1997. Whither Down's syndrome critical regions? *Hum Genet* **99**: 421–423.

Vacik T, Forejt J. 2003. Quantification of expression and methylation of the *Igf2r* imprinted gene in segmental trisomic mouse model. *Genomics* **82**: 261–268.

Copyright © 2003 John Wiley & Sons, Ltd. 
